# The use of 4-Hexylresorcinol as antibiotic adjuvant

**DOI:** 10.1371/journal.pone.0239147

**Published:** 2020-09-22

**Authors:** Y. A. Nikolaev, A. V. Tutel’yan, N. G. Loiko, J. Buck, S. V. Sidorenko, I. Lazareva, V. Gostev, O. Y. Manzen’yuk, I. G. Shemyakin, R. A. Abramovich, J. Huwyler, G. I. El’-Registan

**Affiliations:** 1 Federal Research Centre “Fundamentals of Biotechnology”, Russian Academy of Sciences, Moscow, Russia; 2 Central Research Institute of Epidemiology of Russian Federal Service for Surveillance on Consumer Rights Protection and Human Wellbeing (Rospotrebnadzor) and I.M. Sechenov First Moscow State Medical University of the Ministry of Health of the Russian Federation (Sechenov University), Moscow, Russia; 3 Department of Pharmaceutical Sciences, University of Basel, Basel, Switzerland; 4 Peoples’ Friendship University of Russia (RUDN University), Moscow, Russia; 5 Pediatric Research and Clinical Center for Infectious Diseases, Saint Petersburg, Russia; 6 I.I. Mechnikov North Western State Medical University, St Petersburg, Russia; 7 State Research Center for Applied Microbiology and Biotechnology of Russian Federal Service for Surveillance on Consumer Rights Protection and Human Welfare (Rospotrebnadzor), Obolensk, Russia; Nanyang Technological University, SINGAPORE

## Abstract

Ever decreasing efficiency of antibiotic treatment due to growing antibiotic resistance of pathogenic bacteria is a critical issue in clinical practice. The two generally accepted major approaches to this problem are the search for new antibiotics and the development of antibiotic adjuvants to enhance the antimicrobial activity of known compounds. It was therefore the aim of the present study to test whether alkylresorcinols, a class of phenolic lipids, can be used as adjuvants to potentiate the effect of various classes of antibiotics. Alkylresorcinols were combined with 12 clinically used antibiotics. Growth-inhibiting activity against a broad range of pro- and eukaryotic microorganisms was determined. Test organisms did comprise 10 bacterial and 2 fungal collection strains, including *E. coli* and *S. aureus*, and clinical isolates of *K. pneumoniae*. The highest adjuvant activity was observed in the case of 4-hexylresorcinol (4-HR), a natural compound found in plants with antimicrobial activity. 50% of the minimal inhibitory concentration (MIC) of 4-HR caused an up to 50-fold decrease in the MIC of antibiotics of various classes. Application of 4-HR as an adjuvant revealed its efficiency against germination of bacterial dormant forms (spores) and prevented formation of antibiotic-tolerant persister cells. Using an *in vivo* mouse model of *K. pneumoniae*-induced sepsis, we could demonstrate that the combination of 4-HR and polymyxin was highly effective. 75% of animals were free of infection after treatment as compared to none of the animals receiving the antibiotic alone. We conclude that alkylresorcinols such as 4-HR can be used as an adjuvant to increase the efficiency of several known antibiotics. We suggest that by this approach the risk for development of genetically determined antibiotic resistance can be minimized due to the multimodal mode of action of 4-HR.

## Introduction

Growing inefficiency of known medical preparations due to antibiotic resistance (ABR) of pathogenic microorganisms is a serious problem. [1] Mechanisms of ABR and approaches to minimize (or prevent) these phenomena are therefore a focus of basic research in antibiotic therapy.

ABR may emerge as a result of mutagenesis with subsequent inheritance of genetic determinants, or acquisition of antibiotic resistance determinants via lateral transfer between microorganisms. Overcoming ABR is an important albeit difficult task. [2] Historically, research on suppression of antibiotic resistance and enhancement of the efficiency of antibiotic treatment mainly focused on the search for new antibiotics. However, the number of new antibiotics introduced into medical practice is quite low, with only two new antibiotics registered during the 2000-2013 period. [3] Development of new drug products by combining established or new antibiotics may partially compensate for this insufficient introduction of new compounds. Such combinations may consist of antibiotic hybrids [4] or combinations of two or more antimicrobial agents with different targets in microbial cells to exploit synergistic effects. [5] Alternatively, the effect of an antibiotics can be enhanced by concomitant interference with mechanisms of bacterial resistance. For example, clavulanic acid inhibits beta-lactamases, the enzymes degrading beta-lactam antibiotics, and thus increases the effect of the latter. [6, 7] Substances such as clavulanic acid are adjuvants. They have *per se* no (or only a limited) antibacterial activity but they nevertheless potentiate the effect of antibiotics. [8–11]

Another mechanism of therapy evasion consists of emergence of a small subpopulation of nondividing persister cells in a growing microbial population. [12–18] Persister cells are by definition insensitive (tolerant) to antibiotics. Such cells survive an antimicrobial intervention, germinate after termination of the treatment, and form a new population of antibiotic-sensitive cells. They will have similar properties as the original population and are similarly capable of forming persister cells again. [12–15, 18] The phenotypic transition from a vegetative to a persistent state is associated with development of stress responses and mutations due to low-accuracy reparation of damaged DNA. It should be noted that in the case of resistance-related mutations, this may lead to emergence of genetically determined (inherited) ABR. [19]. More recently, approaches to minimize the formation of persister cells or to prevent germination were introduced. [8, 20–22] Persisters may be targeted by antibiotics or combined antimicrobial preparations either at the stage of their formation, in order to minimize their numbers, [23] or at the stage of their germination, in order to sensitize them to the action of antibiotics and to prevent reverse phenotypic transition. For example, quorum-sensing autoinducer AIA-1 potentiates the therapeutic effect of an antibiotic during treatment of experimental murine *Pseudomonas aeruginosa* infection by decreasing the numbers of antibiotic-tolerant persister cells. [23]

**Fig 1:**
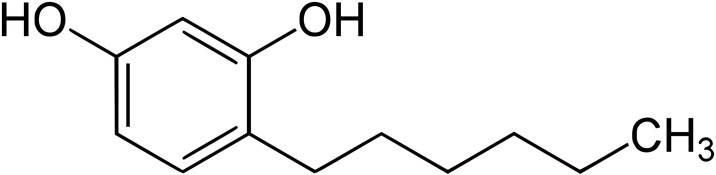
Chemical structure of 4-hexylresorcinol (4-HR). 4-hexylbenzene-1,3-diol has a molecular weight of 194.27 g/mol, a solubiliy in water of 800 mg/L, and an octanol-water partition coefficient of 3.9.

It was the aim of the present study to identify novel antibiotic adjuvants to enhance the effect of various classes of antibiotics. Our work was based on two assumptions: decreased metabolic activity in the cells of pathogenic microorganisms caused by an adjuvant will result in suppressed mechanisms of antibiotic resistance and minimization of the number of surviving persister cells (up to their possible elimination). This will decrease the risk of reinfection and the formation of genetic resistance determinants. Alkylresorcinols were identified to have the required properties. These phenolic lipids are secondary metabolites of plants and can as well be found in some microorganisms. [24] They have a weak antimicrobial activity. 4-hexylresorcinol (4-HR) (Fig 1) is a synthetic derivative used as topical drug in antibacterial oral gargles and throat lozenges. [25] Recently, an incorporation of 4-HR within PLGA polymer composite films was proposed as a strategy to obtain biomedical and packaging products with antimicrobial properties. [25] In food industry, 4-HR is frequently used as a preservative and to prevent browning. [26] The widespread use in food industry and the antiseptic activity of 4-HR give rise to the assumption that 4-HR might be a safe adjuvant for antibiotic combination therapies.

## Materials and methods

### Alkylresorcinols

4-hexylresorcinol (Fig 1) was purchesed from Sigma- Aldrich (St. Louis, MO, USA) while other alkylresorcinols were provided by Carboshale Ltd. (Virumaa, Estonia). Phenolic lipids were added as stock solutions in ethanol (3% vol/vol) or dimethyl-sulfoxide (DMSO) (3% vol/vol).

### Microorganisms

Nonpathogenic strains of pathogenic microorganisms such as gram negative *E. coli* K12 and gram positive *S. aureus* 209P were obtained from the fungal and bacterial collection of the All-Russian Collection of Microorganisms (Pushchino, Moscow Region, Russian Federation, see Table 1). For the chequerboard method and the *in vivo* experiments, the following strains from the collection of the Research Institute on Infant Infections (St. Petersburg, Russia) were used: *Staphylococcus aureus* ATCC 29213 (reference strain) and isolates MRSA *S. aureus* (SA0077, SA0206, SA0411 SA0318); clinical isolates *Enterococcus faecalis* E23 and E28; *Escherichia coli* ATCC 25922 (reference strain) and *Escherichia coli* isolates (1273, 1263); clinical isolates of *Klebsiella pneumoniae* (1191, 204, 895); *Pseudomonas aeruginosa* (1215, 56); *Acinetobacter baumannii* (121, 1308); as well as clinical isolate *Klebsiella pneumoniae* KPM9 from the collection of the State Research Center for Applied Microbiology and Biotechnology (Obolensk, Russia). Bacterial cultures were grown in LB medium for 18-20 hours. Fungal cultures were grown in wort medium for 36 hours (to the stationary growth phase). Second cycle cultures were inocculated and grown for 1 hour to the early exponential phase in 250 mL flasks with 50 mL of LB medium. Samples were dispensed (2 mL) into glass test tubes containing the indicated amounts of antibiotics and adjuvants (phenolic lipids). Starting concentration was 5 x 10^5^ cells/mL. The test tubes were incubated for 24 hours at 30°C on a rotary shaker (100 rpm) to determine the minimal growth-inhibiting concentrations (MIC) of saprophytic organisms or non-pathogenic strains of bacteria (Table 1). Bacterial growth was assessed spectrophotometrically by OD_600_ measurements on a Jenway Spectrophotometer 7315 (Jenway, Staffordshire, UK).

**Table 1:** Effect of 4-hexylresorcinol (4-HR) on a selection of various microorganisms. MIC values are means of two series of experiments.

Classification	Microorganism	Strain No.	MIC (mg/L)
Prokaryota (Gram positive)	*Bacillus subtilis*	VKM-B436	20
Prokaryota (Gram positive)	*Lactococcus lactis*	B-1662	20
Prokaryota (Gram positive)	*Micrococius luteus*	VKM-Ac2230	20
Prokaryota (Gram positive)	*Streptococcus faecalis*	B-602	20
Prokaryota (Gram positive)	*Staphylococcus aureus*	209P	25
Prokaryota (Gram positive)	*Streptomyces coelicolor*	Ac-738	30
Prokaryota (Gram positive)	*Lactobacillus acidophilus*	B-1660	50
Prokaryota (Gram negative)	*Pseudomonas carboxydaflava*	Z-1107	65
Prokaryota (Gram positive)	*Mycobacterium smegmatis*	Ac-1239	70
Prokaryota (Gram negative)	*Escherichia coli*	MC4100	200
Eukaryota	*Aspergillus niger*	F-2039	300
Eukaryota	*Saccharomyces cerevisiae*	Y-375	300

### Determination of antibacterial activity of alkylresorcinols

The tested antibiotics are listed in S1 Table. The degree of prolongation of alkylresorcinol bactericidal action was determined by the use of a mixed culture of gram positive *B. subtilis*, gram negative *P. fluorescens*, and yeasts *S. cerevisiae*. Individual cultures were grown to the stationary phase and mixed in equal amounts to prepare the inoculum. The inoculum (5% vol/vol) was added to the medium consisting of a mixture (1:1) of LB medium and wort. Flasks were incubated and bacterial growth was assessed spectrophotometrically as described above.

### Minimal growth-inhibiting concentrations (MIC)

Antibiotics were characterized by their minimal growth-inhibiting concentrations (MIC), which were determined for every test organism using conventional serial dilution in liquid media followed by plating. Phenolic lipids were used at concentrations of $\frac{1}{2}$ MIC; the latter was determined for each test organism. The criteria for adjuvant efficiency were as follows: decreased MIC of a given antibiotic in combination with AR ($\frac{1}{2}$ MIC), which was determined as the absence of growth of a test organism after 24 hour incubation in liquid medium; decreased number of viable persister cells (colony-forming units, CFU) retaining viability after 24 hour incubation at high antibiotic concentration (10 MIC and above, by [27]). CFU numbers were determined by plating serial ten-fold dilutions on agar (1.5%) LB medium. All values represent means of three independent sets of experiments with three repetitions each.

### Chequerboard method

Stock solutions of the tested antibiotics were prepared to obtain two-fold serial dilutions in vertical rows 1-8 of a 96-well plate. 4-HR stock solutions were prepared to cover final concentrations of 8 to 512 μg/mL. 0.1 mL of antibiotic and/or 4-HR solution in broth (or broth only) was added to a well, then 0.1 mL of bacteria suspension was added to a final concentration of 5 x 10^5^ cells/mL. Cultivation temperature was 35-37°C. Results were assessed by measuring bacterial growth in the vertical and horizontal rows. MIC was defined as the minimal concentration at which bacterial growth was absent. The type of interaction of antibiotics (AB) and 4-HR was calculated using the FICI coefficient (Eq 1) as recommended: [28]

$$\begin{array}{c} FICI=\frac{MIC(AB+4-HR)}{MIC(AB)}+\frac{MIC(AB+4-HR)}{MIC(4-HR)} \end{array}$$ (1)

Apart from the recommended coefficients, one more was introduced (K1), showing the MIC decrease for the monopreparation compared to the binary one:

$$\begin{array}{c} K1=\frac{MIC(AB)}{MIC(AB+4-HR)} \end{array}$$ (2)

With K1 reflecting the decrease in the effective dose of antibiotic in the presence of an ineffective concentration of 4-HR.

### *K. pneumoniae* KPM9 induced experimental sepsis

Outbred female white mice (18-21 g; Andreevka Farm, Scientific Center for Biomedical Technologies, Russia) were infected intraperitoneally by *K. pneumoniae* (clinical isolate KPM9) cell suspensions (0.5 mL, 50 × LD_50_). The LD_50_ value was calculated according to Cerber in the modification by Ashmarin and Vorov’ev [29] according to survival duration after infection. The average LD_50_ values for *K. pneumoniae* KPM9 were 21 cells.

Mice were treated with polymyxin B (Pm; Bharat Sirams and Vaksins Ltd., India) and/or 4-HR. The doses were calculated as recommended. [29, 30] Treatment began 24 hours after infection (day 0). Pm was administered intramuscularly. 4-HR was administered *per os* twice a day for five days as as solution in 50% ethanol using an oral gavage needle (0.2 mL). Mice were monitored for 14 days. Criteria of treatment efficiency were survival and contamination of internal organs with bacteria (S3 Table). Severity of the infection was assessed using the overall condition of the animals (behavior, mobility, appetite, weight, and lymph node size). Surviving animals were euthanized, and bacteriological and postmortem study of their organs was carried out, including plating of the lungs, spleen, and blood samples onto solid HRM medium followed by a 48 hour incubation at 37°C.

### Laboratory animal welfare

Animals were kept under standard conditions according to guidelines of the US National Institutes of Health (NIH Publication No. 8023, revised 1978). Mice had free access to water and food (Laboratorkorm, Moscow, Russia) and were kept in polycarbonate cages (LabProducts, Seaford, DE) in groups of up to 10 animals. Animal health and behavior was monitored twice daily. Animals were handled by animal caretakers, which were trained in accordance to protocols established by the USAMRIID, WRIAR and UHSUS. Animal experiments were approved by the Bioethics Commission, State Research Center for Applied Microbiology & Biotechnology. Animal experiments involved fast spreading mice sepsis with a lethality of 100% without treatment within 5 days. Surviving animals were euthanized after 14 days. There is no alternative to these experiments since only an animal experiment can model all aspects of the bacterial infection [31, 32]. Studies were designed to measure survival percentages. In view of the severity and rapid progression of the infection, animals were bound to die from the disease. This provides a very small window of opportunity to intervene and to decide whether animals have reached a humane end point and should be euthanized. The number of animals was 60, 25 of which were found dead and 35 were euthanized.

## Results

### Antimicrobial activity of alkylresorcinols and phenolic lipids

**Fig 2:**
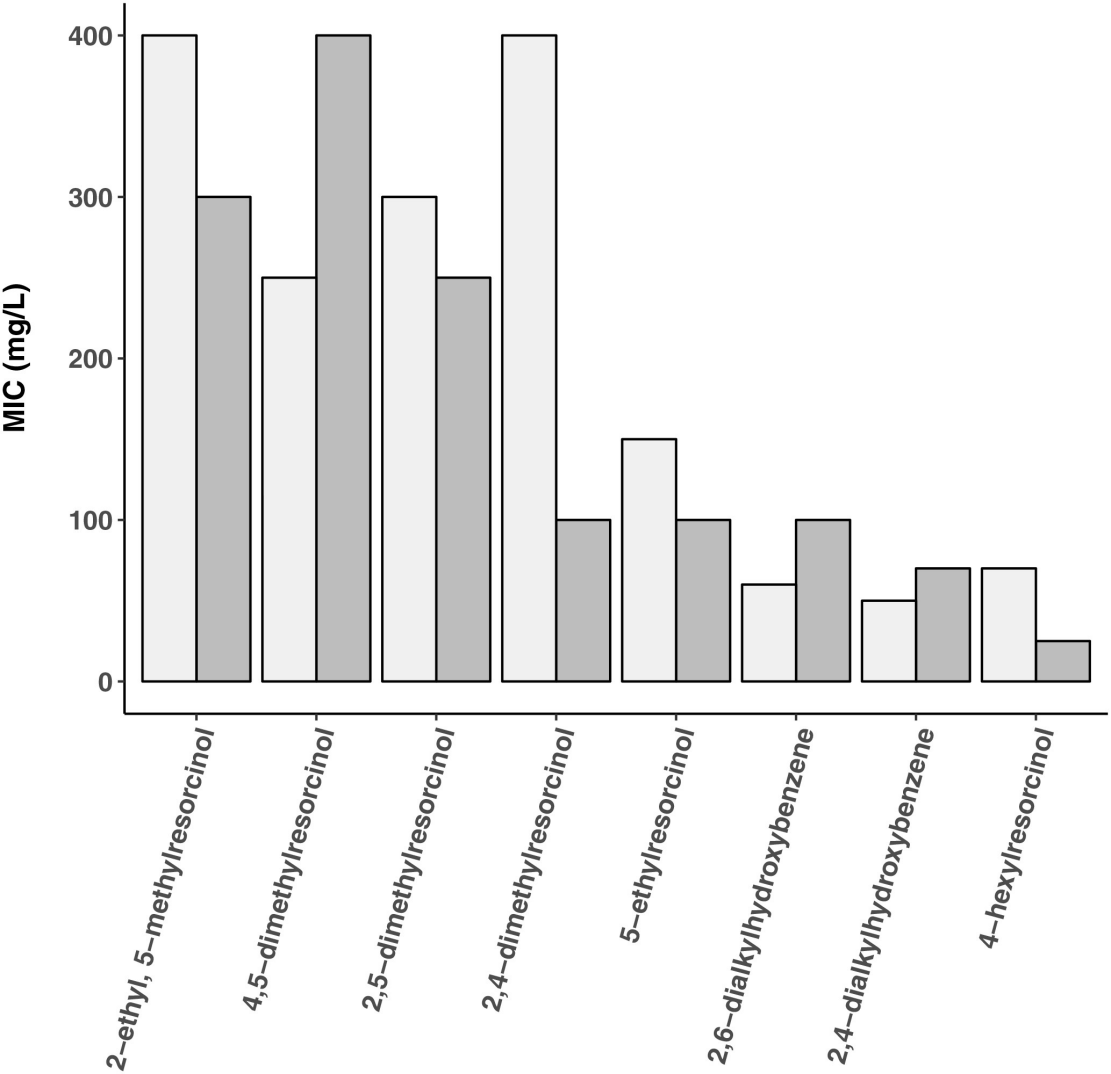
Minimal growth-inhibiting concentrations (MIC) of alkylresorcinols. 5-methylresorcinol showed poor activity against *M. smegmatis* (MIC = 300 mg/L, light grey bars) and *S. aureus* (MIC >5000 mg/L, dark grey bars). Values are means of two independent series of screening experiments.

Based on the hypothesis that phenolic lipids (Figs 1 and 2) can be used as adjuvants for antibiotic therapy, screening experiments were performed to study their impact on bacterial growth (MIC determinations) and subsequently synergistic effects with different classes of antibiotics (S1 Table). The MIC of phenolic lipid preparations were determined using as test organisms *S. aureus* 209P (a nonpathogenic *S. aureus* strain) and *M. smegmatis* AC-1239 (a nonpathogenic analog of the pathogen *M. tuberculosis*) (Fig 2). Different types of alkylresorcinols, amphiphilic 2,4- and 2,6-dialkylhydroxybenzenes and the more hydrophobic 4-hexylresorcinol (4-HR) were tested. In view of the high activity of 4-HR, its antimicrobial activity was subsequently studied using a broad range of microorganisms (Table 1). Gram positive bacteria were found to be most sensitive to 4-HR, with MICs of 20-50 mg/L. *M. smegmatis* was characterized by a MIC of 70 mg/L. Gram negative bacteria, yeasts and fungi were less sensitive.

### Effect of 4-hexylresorcinol as antibiotic adjuvant

In the next series of experiments, MIC was determined for each antibiotic supplemented with 4-HR at the concentration of $\frac{1}{2}$ MIC, which was insignificant for preventing growth of the test organisms, but suppressed their metabolic activity. The results are presented in Tables 2 and 3. In the case of *S.aureus* 209P, the presence of 4-HR ($\frac{1}{2}$ MIC) enhanced the antimicrobial action of all tested antibiotics, especially of polymyxin, vancomycin, capremabol, levomycetin, and ampicillin (5- to 10-fold increase). In the case of *E. coli* K12, a significant intensification of the antimicrobial action was as well observed in presence of 4-HR ($\frac{1}{2}$ MIC). 4-HR had the greatest effect on activity of polymyxin, gentamicin, azithromycin, and levomycetin (MIC decreased 10- to 50-fold). The lowest effect (2- to 3-fold) was observed for cyclosporine, vancomycin, and rubomycin. In the case of a yeast-like organism (i.e. *C. utilis*), two antibiotics were tested: ciprofloxacin and naftifine hydrochloride. However, their MIC decreased insignificantly in presence of 4-HR. Antimicrobial action of the studied antibiotics against both tested bacteria increased in the presence of $\frac{1}{2}$ MIC of 4-heptylresorcinol leading to similar albeit weaker effects as observed with 4-HR. In view of the higher potency of 4-HR, only this compound was used for the next series of experiments.

**Table 2:** MIC of various antibiotics against gram positive *S. aureus*. MIC of antibiotics alone or antibiotics combined with 15 mg/L 4-HR corresponding to $\frac{1}{2}$ MIC are shown. Values are means of two series of experiments.

Antibiotic	MIC antibiotic (μg/L)	Fold decrease of MIC in presence of 4-HR
Capremabol	40	10
Ampicillin	0.25	5
Vancomycin	0.5	5
Levomycetin	2.5	5
Polymyxin	10	5
Gentamicin	0.2	4
Doxycycline	0.4	4
Rubomycin	200	4
Ciprofloxacin	3	3

**Table 3:** MIC of various antibiotics against gram negative *E.coli*. MIC of antibiotics alone or antibiotics combined with 100 mg/L 4-HR corresponding to $\frac{1}{2}$ MIC are shown. Values are means of two series of experiments.

Antibiotic	MIC antibiotic (μg/L)	Fold decrease of MIC in presence of 4-HR
Polymyxin	5	50
Gentamicin	1	10
Azithromycin	1	10
Levomycetin	12.5	10
Ciprofloxacin	0.75	5
Doxycycline	5	5
Capremabol	100	5
Ampicillin	40	4
Tobramycin	60	4
Rubomycin	300	3
Vancomycin	200	2

### Effect of 4-hexylresorcinol on germination

Dormant microbial forms lack metabolic activity and therefore targets of many biocidal agents. Moreover, they exhibit high resistance to damaging factors. Suppression of their germination is therefore a challenge. Addition of 4-HR for the indicated period of time to liquid medium prior to inoculation inhibited germination of *B. cereus* spores (Table 4). The decrease in the titer of viable spores (CFU) depended both on 4-HR concentration and exposure duration. We conclude that 4-HR exhibits antimicrobial activity against both bacterial vegetative cells and their dormant forms. Pretreatement of spores impairs their ability to germinate.

**Table 4:** Impact of 4-hexylresorcinol on germination of *B. cereus* spores. Liquid medium containing *B. cereus* B-504 spores was pretreated with 4-HR for the indicated period of time. Germination was in absence ((-); 100% control without 4-HR corresponds to 6.0 ± 1.0 x 10^7^ CFU/mL) or presence ((+); 100% control without 4-HR corresponds to 9.5 ± 0.5 x 10^7^ CFU/mL) of 4-HR. Values are means of two series of experiments.

Treatment	4-HR (mg/L)	% rate of germination
No pretreatment (+)	5	95 ± 15%
	10	77 ± 13%
	25	0%
1 hour pretreatment (-)	50	100 ± 18%
	100	98 ± 16%
	500	83 ± 14%
	1000	51 ± 11%
2 days pretreatment (-)	50	75 ± 16%
	100	75 ± 14%
	500	55 ± 11%
	1000	20 ± 3%

### Effect of 4-hexylresorcinol on persister cells

4-HR was combined with antibiotics to study the effect of 4-HR on viability of persister cells, i.e. cells remaining viable in the presence of biocidal concentrations of antibiotics [18]. The test strain *E. coli* K12 was treated with ampicillin and ciprofloxacin (antibiotics inhibiting different targets in bacterial cells, i.e. cell wall synthesis and DNA synthesis, respectively). Their concentrations of 60 and 100 μg/mL, respectively, was above their MIC. The results presented in Table 5 demonstrate a decrease in the numbers of surviving persister cell in presence of 4-HR ($\frac{1}{2}$ MIC). No viable cells were found after 2 days (for ampicillin) and 7 days (for ciprofloxacin) if antibiotics were combined with 4-HR. A neglegible amount of viable cells (i.e. 32 CFU/ml) was detected after 1 day of incubation with ampicillin in the presence of 4-HR. This is in contrast to incubations with the antibiotics alone, which did not lead to complete eradication of the microorganisms under all tested conditions (viable cell count was >3 x 10^3^ CFU/mL).

**Table 5:** Effect of 4-HR on persister cells. CFU of *E.coli* were determined after incubation for the indicated period of time with biocidal antibiotic concentrations (60 μg/mL of ampicillin and 100 μg/mL of ciprofloxacin) in presence and absence of 4-HR ($\frac{1}{2}$ MIC). Initial cell titer was 10^9^ CFU/mL. Values indicate fold change as compared to control (absence of antibiotics). >10^6^: <500 remaining CFU/mL detected. Values are means ± SD, n = 3.

Antibiotic	Incubation	Fold reduction w/o 4-HR	Fold reduction with 4-HR
Ampicillin	3 hours	40	900’000
Ampicillin	24 hours	125	>10^6^
Ampicillin	48 hours	4’000	>10^6^
Ciprofloxacin	3 hours	40	200’000
Ciprofloxacin	48 hours	1’600	>10^6^
Ciprofloxacin	168 hours	300’000	>10^6^

### Effects of 4-HR on clinical isolates of pathogenic bacteria

Synergistic effects of antibiotics and 4-HR were studied using clinical isolates of pathogenic bacteria causing infections associated with medical interventions. The chequerboard method was applied to calculate a fractional inhibitory concentration index (FICI). [28] FICI values were categorized into three groups: Synergy (FICI ≤ 0.5), indifference, or absence of interaction, (0.5 ≤ FICI ≤ 4.0), and antagonism (FICI ≥ 4.0). Additionally, the coefficient K1 was calculated. With this coefficient, the decrease in the effective dose of antibiotic in the presence of 4-HR (K1) can be determined.

For this assessment, clinical isolates of gram positive bacteria (*S. aureus* and *E. faecium*) as well as gram negative bacteria (*E. coli*, *K. pneumoniae*, *A. baumannii*, *P. aeruginosa*) were selected (S2 Table). In 26% of the studied antibiotic with 4-HR combinations, no effect of 4-HR on the MIC of antibiotics was observed. But in the remaining 74%, the MIC of the studied antibiotics decreased 2- to 512-fold, with a fourfold and more pronounced MIC decrease found in 33% of the combinations and an eightfold and more pronounced MIC decrease occurred in 12% of the groups. Importantly, potentiation of antibiotic activity (strong synergistic effects as indicated by the FICI parameter and decrease in their effective doses in combination with 4-HR as indicated by the K1 parameter) was observed for both sensitive and resistant strains. 4-HR exhibited synergism and caused a significant decrease in the MIC of antibiotics in the case of interactions with ciprofloxacin (7 combinations), polymyxin (5 combinations), and amikacin (4 combinations). 4-HR efficiency varied depending on microbial taxa and was higher in the case of gram negative bacteria. 4-HR in combination with polymyxin showed synergism against *A. baumannii*, *P. aeruginosa* and *K. pneumoniae* with different levels of sensitivity. As a representative example, results for clinical isolates of *K. pneumoniae* KPM9 (covering both sensitive and resistant strains) are shown in Table 6. In the case of polymyxin, for example, strong synergistic effects were observed (FICI = 0.07 μg/L) with a 16-fold decrease in the effective dose of antibiotic in the presence of 4-HR.

**Table 6:** Assessment of interactions between 4-HR and various groups of antibiotics against clinical isolates of gram negative pathogenic *K. pneumoniae*. The chequerboard method yields FICI values (Eq 1) as well as the coefficient K1 (decrease in the effective dose of antibiotic in the presence of 4-HR; Eq 2). Synergy is defined by FICI ≤ 0.5, absence of interaction by 0.5 ≤ FICI ≤ 4.0, and antagonism by FICI ≥ 4.0. For the resistant pathogen, 4-HR concentrations were 512 μg/L. For the sensitive strain, 4-HR concentrations were 64 μg/L. Unit of MIC is μg/L. MIC AB designates MIC of the antibiotic alone.

Antibiotic	Phenotype	MIC AB	K1	FICI
Amikacin	Sensitive	1	1	2
Ampicillin	Sensitive	8	0.25	5
Cefotaxim	Sensitive	0.5	2	1.5
Ciprofloxacin	Sensitive	0.06	4	0.26
Meropenem	Sensitive	0.03	4	0.26
Polymyxin	Sensitive	0.25	16	0.07
Tigecycline	Sensitive	0.12	1	2
Amikacin	Resistant	128	16	0.09
Ampicillin	Resistant	256	0.1	8.06
Cefotaxim	Resistant	128	8	0.25
Ciprofloxacin	Resistant	128	16	0.09
Meropenem	Resistant	16	2	0.56
Tigecycline	Resistant	2	2	0.56

### *In vivo* proof of concept using an experimental mouse sepsis model

**Fig 3:**
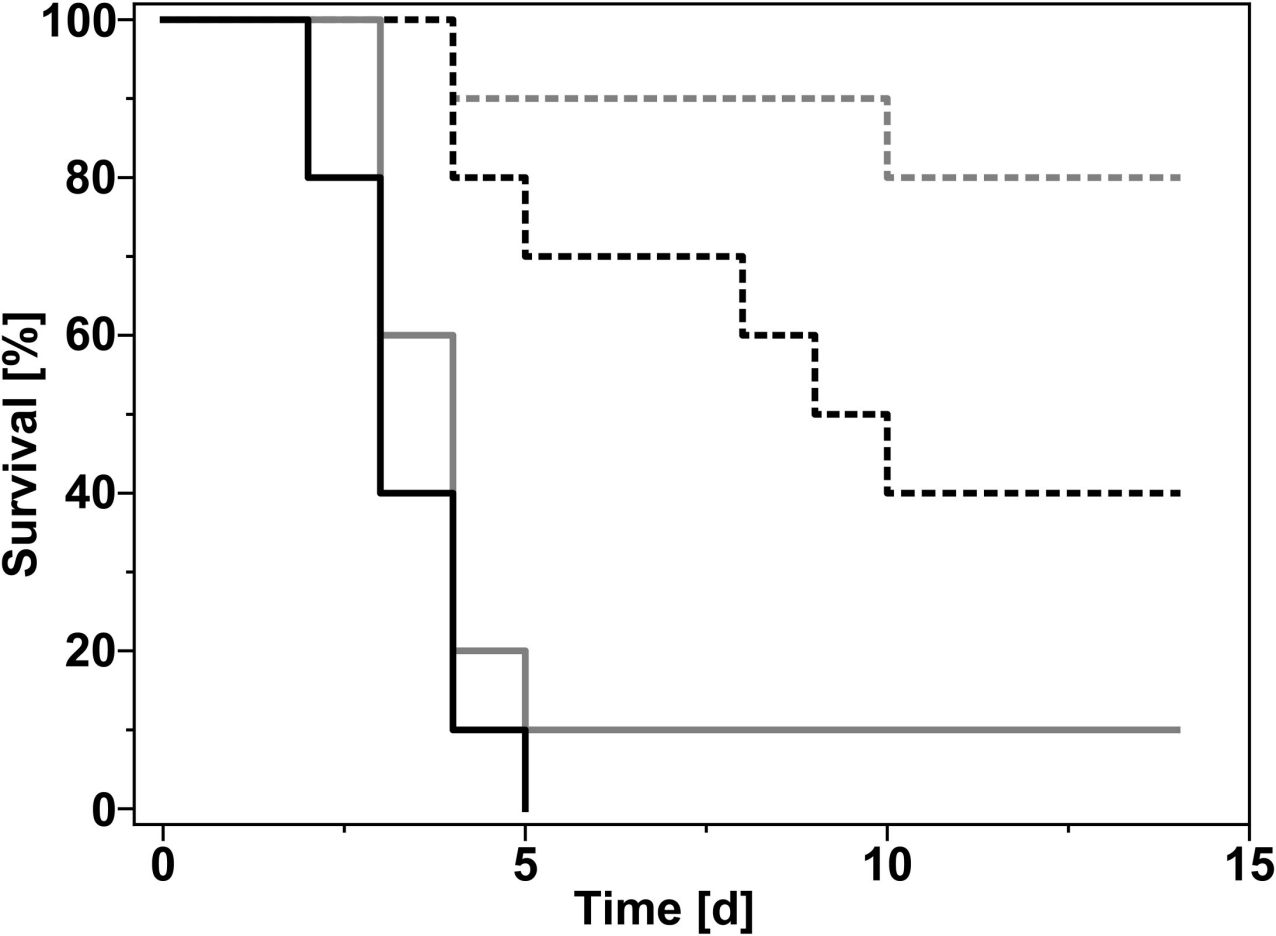
Effect of 4-hexylresorcinol in a mouse bacterial sepsis model. A total of 10 mice per group were infected with *K. pneumoniae* (clinical isolate KPM9; 1000 CFU/mouse). Treatment started 24 hours after infection (day 0). Mice were treated with polymyxin (1 mg/kg, intramuscular) and/or 4-HR (15 mg/kg twice a day for five days, *per os*). Dashed grey line: polymyxin and 4-HR combined. Dashed black line: polymyxin alone. Solid grey line: 4-HR alone. Solid black line: untreated control.

Based on the results of the chequerboard analysis (Table 6), a mouse experimental sepsis model was developed to demonstrate *in vivo* effects of a combination of polymyxin and 4-HR in infections caused by clinical isolates of pathogenic *K. pneumoniae*. For our model of mouse klebsiellosis, ten outbred mice per group were infected by intraperitoneal injection of a culture of antibiotics sensitive *K. pneumoniae* KPM9 (0.5 mL, ≈ 50 LD_50_, 1000 CFU/mouse). Treatment of groups started 24 hours after infection and continued for five days. Since oral polymyxin (Pm) preparations are not well tolerated in mice, Pm was applied as intramuscular injection (1 mg/kg). The 4-HR preparation was administered twice a day *per os* (PO). Total daily 4-HR doses were 30 mg/kg. A representation of the survival data for Pm in combination with 4-HR is shown in Fig 3. On day 14 of the experiment, surviving animals were euthanized. Their spleen, blood, and lungs were examined to determine viable cell titers (CFU) of *K. pneumoniae* by plating serial dilutions on solid media.

Indications of the *Klebsiella* infection (adynamia, anorexia, and loss of weight) were observed during the experiment and resulted in a decrease in the body mass as well as death of animals. Treatment of animals with the Pm + 4-HR combination showed high efficiency when the 4-HR preparation was administered *per os*. Survival rate in the group treated with Pm + 30 mg/kg/day 4-HR was the highest (80%), while all the mice in the control group died. Moreover, life duration (9 days) of animals treated with Pm + 30 mg/kg/day 4-HR was more than twice that of the control mice (4 days). Approximately 75% of surviving animals in this group were sanified, as demonstrated by an absence of viable *K. pneumoniae* in inner organs. In the remaining mice, bacterial numbers in the spleen and lungs were approximately 3.2×10^2^ and approximately 5.4×10^2^ CFU/mL, respectively (S3 Table). No bacteria were found in the blood. Surprisingly, survival rates actually decreased to 60% in groups treated with higher 4-HR doses (i.e. 50 or 75 mg/kg/day). Higher doses of 4-HR did as well reduce the percentage of sanified mice (40% and 30% of surviving mice treated with 50 and 75 mg/kg/day, respectively). It should be noted that intraperitoneal administration of 4-HR was abandoned since it showed low efficiency and caused pain. Treatment of animals with 1 mg/kg Pm alone did not result in complete recovery of mice: in 40% of surviving animals, inner organs were contaminated with *K. pneumoniae*. None of the surviving animals receiving 4-HR alone were sanified.

## Discussion

Research on microbial growth autoregulation revealed microorganisms (both pro- and eukaryotic) to produce low-molecular mass compounds acting as density regulators, which in a number of bacteria are phenolic lipids of the alkylresorcinol group. [33] Alkylresorcinols were subsequently shown to act as autoinducers of the quorum-dependent regulatory system. [33, 34] This is in agreement with literature confirming this function of alkylresorcinols. [35] At increasing concentrations of alkylresorcinol, population growth of microorganisms is restricted and the development of a hypometabolic and then anabiotic state is induced. [33, 34] This motivated us in the present study to explore a possible role of alkylresorcinols and other phenolic lipids as antibiotic adjuvants.

4-HR (4-hexylresorcinol) was identified in screening experiments to have the strongest effects on bacterial growth. In these experiments, antimicrobial activity of alkylresorcinols with methyl-, ethyl-, and hexyl groups was found to increase with increasing hydrophobicity. Comparison of 4-HR and 2-ethyl-5-methylresorcinol revealed that the absence of screening of hydroxyl groups by a large alkyl group was important for antimicrobial activity. Thus, while the more hydrophobic compound 2-ethyl-5-methylresorcinol could have been expected to have higher antimicrobial activity than 4-HR, it was in fact less toxic to the test organisms. Comparison of 2,4- and 2,5-dimethylresorcinols revealed hydroxyl group screening to be less important for *S. aureus* (MIC 100 and 250 mg/L, respectively) than to *M. smegmatis* (400 and 300 mg/L, respectively).

Antimicrobial activity of 4-HR on microorganisms seem to depend on cell wall properties. High sensitivity was associated with a peptidoglycan-based outer cell wall structure of gram positive bacteria. The additional outer lipid membrane of gram negative bacteria, as well as the complex chitin and polysaccharide-based cell wall structure of yeast and fungi, seems to confer some degree of resistance. High resistance to 4-HR in *M. smegmatis* might be due to the presence of lipid structures and mycolic acids, causing high hydrophobicity of their cell surface. Dependence of antimicrobial activity of phenolic lipids on their hydrophobicity is in agreement with literature, [36] which reports increased antimicrobial activity of n-alkylphenols with increasing length of the alkyl chain and higher resistance of microorgansisms with lipid-enriched cell membranes to alkylphenols. Based on these results, 4-HR was selected for further characterization.

The utility of 4-HR as potent adjuvant for the treatment of bacterial infections was demonstrated in combination with several antibiotics. The biocidal effects of these antibiotics to *S. aureus* and *E. coli* were potentiated 5- to 50-fold in the presence of $\frac{1}{2}$ MIC 4-HR. This result opens the opportunity for novel treatment regimens against bacterial infections. For instance, a higher bactericidal effect obtained with the combination of antibiotic and 4-HR could result in lower doses of antibiotics to be applied to a patient while still maintaining the same effectiveness. Consequently, antibiotics that are rarely used in humans due to side effects associated with bactericidal doses (such as polymyxin) might become a viable alternative when combined with 4-HR. In view of the challenges related to antibiotics resistance, this strategy could help to delay the hypothesized post-antibiotics era.

In addition to vegetative forms, bacteria can undergo transformation into a dormant form, leading to the emergence of persister cells. These dormant forms or endospores are characterized by the absence of metabolic activity and therefore possess a high resistance towards environmental insults including radiation, temperature, and chemicals. Previous studies [12, 37–39] suggest that persister cells are responsible for the chronic nature of many bacterial infections and that their formation is induced by prolonged use of antibiotics. [40, 41] This is due to selective pressure on the pathogens promoting persistence. [42, 43] Efficiency of antibiotic therapy can therefore be compromised by the development of antibiotic-resistant clones. Furthermore, persister cells surviving antibiotic treatment may transfer genetic information, e.g. plasmid encoded resistance genes, vertically from parent to offspring or transfer genetic information to another species of bacteria within the same generation by means of transduction, transformation and conjugation (horizontal gene transfer). It is therefore important to eliminate antibiotics-insensitive, dormant persister cells. Based on these considerations, we measured the synergisitc effect of a combination therapy using antibiotics-resistant persister cells. It was indeed possible to demonstrate that the addition of 4-HR significantly decreases the number of germinating *B. cereus* spores in liquid medium, as well as on agar medium. This in contrast to treatment with antibiotics alone. The observed effect was dependent on 4-HR concentration and duration of exposure. Moreover, the number of viable spores after prolonged treatment (14 days) was below the limit of detection. Thus, the risk of reemergence of an infection after termination of the antibiotic treatment due to germination of persister cells can be minimized. This result demonstrates that bacterial strains, that already acquired ABR, can become sensitive to the antibiotic compound again when 4-HR is co-administered. It is therefore tempting to speculate that antibiotics, that are rarely used due to widespread resistance towards that antibiotic, can become a viable treatment alternative again.

To systematically study interactions of 4-HR and antibiotics with clinical isolates of pathogenic bacteria, we used the chequerboard method. A FICI parameter of ≤ 0.5 [28] is thereby indicative of synergism. This classification is confirmed if the MIC of the antibiotic decreases at least fourfold in the presence of 4-HR (K1 ≥ 4). Strong synergism was observed both for gram negative and gram positive bacteria. 4-HR combinations with fluoroquinolones, polymyxin, and amikacin with gram negative bacteria as targets were the most promising variants S2 Table. Interestingly, 4-HR may have both synergistic as well as antagonistic effects. These results emphasize the importance of FICI values and the coefficient K1, which have to be determined for each new combination of antibiotic, 4-HR and pathogen. Importantly, 4-HR enhanced antibiotic activity equally well in both antibiotic sensitive and resistant bacterial strains.

*In vitro* efficiency of antibiotics in 4-HR binary compositions was determined for the gram negative bacterium *Klebsiella pneumoniae*, which causes various forms of klebsiellosis infections. Death rate in humans with the generalized klebsiellosis infection may be as high as 60%. Emergence of antibiotic-resistant *K. pneumoniae* strains is a major problem. [44] Therapy of nosocomial infections is often difficult due to high resistance of the causative agents to most known antibiotics, which develops in 30-50% of the patients in the course of monotherapy. Since the virulence of *K. pneumoniae* depends significantly on its ability to form a polysaccharide capsule preventing phagocytosis, the hypermucoid strain *K. pneumoniae* KPM9 was selected for the development of an animal model. *In vitro* results showed a significant effect of 4-HR on antibiotic efficiency with a FICI value of 0.07 and a 16-fold decrease of the polymyxin MIC in combinations with 4-HR (Table 6). In view of these pronounced synergistic effects, we decided to focus for the following series of experiments on combinations of *K. pneumoniae*, polymyxin, and 4-HR. With respect to selection of the antibiotic, it should be noted that previous *in vitro* studies reported synergistic effects of polymyxin with other antibiotics in that bactericidal activity was enhanced and bacterial heteroresistance was suppressed. [45–47] Clinical studies revealed that a combination therapy including polymyxin has a better clinical outcome as compared to a monotherapy. This can be attributed to a decreased risk of Pm associated side effects and offers interesting options with respect to the use of adjuvants such as 4-HR. [48]

Klebsiellosis treatment is often based on an assessment of the activity of preparations in treatment of experimental *Klebsiella* infections in animals. Mice are highly sensitive to this infection. Mouse klebsiellosis is characterized by an acute generalized disease, with *K. pneumonia* and sepsis in virulent forms. Death due to infection-related toxic shock occurs within a week after infection. Successful antibiotic treatment results in elimination of the infectious agent and normalization of clinical laboratory parameters. In the present study, it was indeed possible to eradicate *K. pneumoniae* in a mouse sepsis model by a combination of polymyxin and 4-HR. Combined therapy resulted in 80% survival rate compared to 40% for the polymyxin monotherapy. Interestingly, 4-HR doses of 30 mg/kg/day were in our experiments more effective than higher doses of 50 or 75 mg/kg/day. It is tempting to speculate that side-effects due to overdosing of 4-HR had a negative impact on the recovery of infected animals. Bacteriological analysis of parenchymatous organs of the animals euthanized 9 days after the end of combined treatment revealed absence of *K. pneumoniae* KPM9 cells in 75% of surviving animals, while all animals treated with the antibiotic alone were still carriers of the infection. Thus, the antibacterial preparation containing 4-HR suppressed formation of persister cells. We therefore propose that 4-HR might be an interesting alternative to sulfonamides and tetracyclines presently used as adjuvants for the treatment of klebsiellosis in humans.

The mechanism of action of 4-HR is polymodal and complex. Similar to other alkylresorcinols, the biological activity of 4-HR may be attributed to its action as a structural modifier of biopolymers and supramolecular structures such as membranes. [33, 34, 36, 49, 50] The amphiphilic nature of 4-HR seems to be responsible for its partitioning into and diffusion across cell membranes. Unlike cationic peptides, however, membrane integrity is not impaired by 4-HR. [51, 52] It has therefore been proposed that formation of complexes with macromolecules and membrane lipids results in increased microviscosity of the membrane lipids, which causes inhibition of their functional activity. [33, 36, 53] This membranotropic action of 4-HR explains the high efficiency of its combinations with the following antibiotics: polymyxin (affecting membranes of gram negative bacteria), fluoroquinolones (inhibiting the activity of helicases located at the initial site of DNA replication), and amikacin, the effect of which depends on the transmembrane potential of bacterial cells. Besides its actions on membranes, 4-HR interacts with DNA [54] and proteins. The latter was demonstrated by studies using enzymes and immune system proteins as models. Effects were dose-dependent and linked to protein conformation and thus functional activity. [34, 50, 55, 56] Increased intracellular 4-HR concentrations induce a stress response leading to generation of reactive oxygen species (ROS) and RpoS (RNA polymerase, sigma factor S) activation. [57]

Due to their unspecific mode of action, 4-HR (and other alkylresorcinols) have unique properties in that they act on bacteria as well as on protozoans and helminths. [36] The outer membrane of gram negative bacteria seems to be the first target for 4-HR, which enables it to affect such forms of intracellular persistence as SCV-variants [58] and L-forms, [59] and to accumulate in phagocytes. [60] Model experiments *in vitro* revealed HR effect on biofilm formation and disintegration of mature biofilms. [61] It is tempting to speculate that the multimodal mode of action of alkylresorcinols prevents habituation or resistance. In fact, 4-HR-induced changes in microbial cells are similar to those observed under low-temperature stress (increased membrane viscosity and decreased enzymatic activity). Thus, adaptation of microbial cells both to low temperature and to elevated levels of alkylresorcinols is unlikely.

## Conclusion

Alkylresorcinols are widespread in nature and are natural compounds present in large amounts in agricultural products (up to 1.5 g/kg in grain). Their daily consumption with food is 10-20 mg and is not considered harmful. [62] Alkylresorcinols are therefore considered to be bioactive nutrition components, [63] which are part of human metabolism without having negative consequences. [64] In experiments with mice, hexylresorcinol doses of up to 125 mg/kg were shown to have no carcinogenic properties. [65] Moreover, they exhibited antimutagenic [66] and antitumor properties. [67, 68] Consequently, 4-HR obtained regulatory authorization to be used for medical applications in human, [65] as well as in cosmetics and food industry. [69, 70] This status as safe compounds is an important advantage of alkylresorcinols, including 4-HR, and paves the way towards their clinical application as proposed antibiotic adjuvants. Our findings suggest that this strategy will allow for a more efficient use of established antibiotics and thereby reduce the risk of bacterial resistance.

## Supporting information

S1 Table Antibiotics used in the present study. (PDF)

S2 Table Interactions between 4-HR and various groups of antibiotics against clinical isolates of pathogenic gram negative bacteria. (PDF)

S3 Table Actual numbers of K. pneumoniae KPM9 cells in organs (cells/mL) from euthanized mice. Organs were analyzed 14 days after inoculation and treatment with the indicated doses of Polymixin B (PmB) and 4-HR for 5 days. For each organ, the range of bacterial counts in contaminated surviving animals (cells/mL) is indicated. Numbers in parenthesis: number of contaminated surviving animals / number of total surviving animals. Total number of animals was 10 per group. A ratio of 1/8 would thus describe a situation where 8 out of 10 mice did survive. From these 8 surviving animals, the respective organ of only one animal was still contaminated.
(PDF)
