# Supplementary material for: The use of 4-Hexylresorcinol as antibiotic adjuvant
Source: PLoS One. 2020 Sep 22;15(9):e0239147. doi: 10.1371/journal.pone.0239147 (PMC7508414; doi:10.1371/journal.pone.0239147)
Supplement: S1 Table — (PDF) [file pone.0239147.s001.pdf]

S1 Table. Antibiotics used in the present study.

Name	Chemical group	Mechanism of action
Albucid	Sulfanilamide	Impairs biosynthesis of tetrhaydrofolate, required for purine and pyrimidine synthesis. Active against Gram-positive and Gram-negative bacteria and fungi.
Ampicillin	Beta-lactam	Penicillin analog, affects cell wall synthesis. Active against Gram-positive and Gram-negative bacteria.
Azithromycin	Macrolide	Suppresses protein synthesis, binding to the 50S ribosome subunit, suppresses peptide translocase at the translation stage. Active against Gram-positive and Gram-negative bacteria.
Capremabol	Polypeptide	Inhibitor of bacterial protein synthesis at the ribosome level. Active agains Gram-positive bacteria (including mycobacteria).
Ciprofloxacin	Quinolone	Impairs DNA synthesis by inhibition of DNA gyrase. Active against Gram-positive and Gram-negative bacteria.
Doxycycline	Tetracycline	Polyketide, a tetracycline analog. Affects protein synthesis by impairing aminoacyl-tRNA binding to the ribosomal A site. Active against Gram-positive and Gram-negative bacteria.
Gentamicin	Aminoglycoside	Affects protein synthesis by impairing ribosomal 50S subunit function. Active against Gram-positive and Gram-negative bacteria.
Levomycetin	Nitrophenol derivative	Impairs protein synthesis by blocking peptidyl transferase activity. Binds to 23S rRNA of the 50S ribosome subunit. Active against Gram-positive and Gram-negative bacteria.
Naftifine	Phenol derivative	Allylamine antifungal agent. Inhibits squalene epoxidase, which results in suppressed biosynthesis of ergosterol, an essential component of fungal cell membranes. Active against fungi.
Polymyxin	Polypeptide	Impairs the structure and function of the cytoplasmic membrane. Active against Gram-negative bacteria.
Rubomycin	Polyheterocycle	Impairs DNA and RNA synthesis. Active against Gram-positive bacteria.
Tobramycin	Aminoglycoside	Affects protein synthesis impairing ribosomal 50S subunit function. Active against Gram-positive and Gram-negative bacteria.
Vancomycin	Glycopeptide	Inhibits cell wall biosynthesis, changes cell membrane permeability, affects RNA synthesis. Active against Gram-positive bacteria (except mycobacteria).
