# Supplementary material for: The use of 4-Hexylresorcinol as antibiotic adjuvant
Source: PLoS One. 2020 Sep 22;15(9):e0239147. doi: 10.1371/journal.pone.0239147 (PMC7508414; doi:10.1371/journal.pone.0239147)
Supplement: S2 Table — (PDF) [file pone.0239147.s002.pdf]

S2 **Table. Interactions between 4-HR and various groups of antibiotics against clinical isolates of pathogenic gram negative bacteria.**

**Pathogen**	**Phenotype**	**Antibiotic**	**K1**	**FICI**
A. baumanii	Sensitive	Amikacin	8	0.37
	Sensitive	Cefoperazone/Sulbactam	8	0.25
	Sensitive	Ciprofloxacin	4	0.5
	Sensitive	Meropenem	8	0.37
	Sensitive	Polymyxin	16	0.125
	Sensitive	Tigecycline	8	0.135
E.coli	Sensitive	Amikacin	4	2.25
	Sensitive	Ampicillin	0.13	9
	Sensitive	Cefotaxim	2	1.5
	Sensitive	Ciprofloxacin	16	0.12
	Sensitive	Meropenem	2	2.5
	Sensitive	Polymyxin	1	1.25
	Sensitive	Tigecycline	2	2.5
P. aeruginosa	Sensitive	Amikacin	8	1.125
	Sensitive	Ceftazidim	4	2.25
	Sensitive	Ciprofloxacin	4	1.25
	Sensitive	Meropenem	4	1.24
	Sensitive	Polymyxin	16	0.09
A. baumanii	Resistant	Amikacin	1	1.5
	Resistant	Cefoperazone/Sulbactam	4	0.75
	Resistant	Ciprofloxacin	1	1.5
	Resistant	Meropenem	4	0.75
	Resistant	Polymyxin	13	0.03
	Resistant	Tigecycline	16	0.125
E.coli	Resistant	Amikacin	1	2.5
	Resistant	Ampicillin	0.03	32.5
	Resistant	Cefotaxim	8	0.625
	Resistant	Ciprofloxacin	4	0.75
	Resistant	Meropenem	1	3
	Resistant	Polymyxin	4	0.75
P. aeruginosa	Resistant	Amikacin	2	2.5
	Resistant	Ceftazidim	16	0.09
	Resistant	Ciprofloxacin	512	0.017
	Resistant	Meropenem	2	1.5
	Resistant	Polymyxin	8	0.09
