# Supplementary material for: The use of 4-Hexylresorcinol as antibiotic adjuvant
Source: PLoS One. 2020 Sep 22;15(9):e0239147. doi: 10.1371/journal.pone.0239147 (PMC7508414; doi:10.1371/journal.pone.0239147)
Supplement: S3 Table — Organs were analyzed 14 days after inoculation and treatment with the indicated doses of Polymixin B (PmB) and 4-HR for 5 days. For each organ, the range of bacterial counts in contaminated surviving animals (cells/mL) is indicated. Numbers in parenthesis: number of contaminated surviving animals / number of total surviving animals. Total number of animals was 10 per group. A ratio of 1/8 would thus describe a situation where 8 out of 10 mice did survive. From these 8 surviving animals, the respective organ of only one animal was still contaminated. (PDF) [file pone.0239147.s003.pdf]

S3 **Table. Actual numbers of K. pneumoniae KPM9 cells in organs (cells/mL) from euthanized mice.** Organs were analyzed 14 days after inoculation and treatment with the indicated doses of Polymixin B (PmB) and 4-HR for 5 days. For each organ, the range of bacterial counts in contaminated surviving animals (cells/mL) is indicated. Numbers in parenthesis: number of contaminated surviving animals / number of total surviving animals. Total number of animals was 10 per group. A ratio of 1/8 would thus describe a situation where 8 out of 10 mice did survive. From these 8 surviving animals, the respective organ of only one animal was still contaminated.

| **Treatment** | **Blood** | **Spleen** | **Lung** |
|---|---|---|---|
| 1 mg/kg PmB + 30 mg/kg 4-HR | 0 (0/8) | 320 (1/8) | 460-620 (2/8) |
| 1 mg/kg PmB + 50 mg/kg 4-HR | 80-220 (2/6) | 290-3200 (2/6) | 17000-53000 (2/6) |
| 1 mg/kg PmB + 75 mg/kg 4-HR | 0 (0/6) | 10 (1/6) | 50-290 (3/6) |
| 1 mg/kg PmB | 40 (1/4) | 20-190 (2/4) | 70-2100 (2/4) |
| 50 mg/kg 4-HR | 0 (0/4) | 50 (1/4) | 10 (1/4) |
